# L-3,3’,5-triiodothyronine and pregnenolone sulfate inhibit *Torpedo* nicotinic acetylcholine receptors

**DOI:** 10.1371/journal.pone.0223272

**Published:** 2019-10-04

**Authors:** Steven X. Moffett, Eric A. Klein, Grace Brannigan, Joseph V. Martin

**Affiliations:** 1 Center for Computational and Integrative Biology, Rutgers University—Camden, Camden, New Jersey, United States of America; 2 Department of Biology, Rutgers University—Camden, Camden, New Jersey, United States of America; 3 Department of Physics, Rutgers University—Camden, Camden, New Jersey, United States of America; University of Sydney, AUSTRALIA

## Abstract

The nicotinic acetylcholine receptor (nAChR) is an excitatory pentameric ligand-gated ion channel (pLGIC), homologous to the inhibitory γ-aminobutyric acid (GABA) type A receptor targeted by pharmaceuticals and endogenous sedatives. Activation of the GABA_A_ receptor by the neurosteroid allopregnanolone can be inhibited competitively by thyroid hormone (L-3,3’,5-triiodothyronine, or T3), but modulation of nAChR by T3 or neurosteroids has not been investigated. Here we show that allopregnanolone inhibits the nAChR from *Torpedo californica* at micromolar concentrations, as do T3 and the anionic neurosteroid pregnenolone sulfate (PS). We test for the role of protein and ligand charge in mediated receptor inhibition by varying pH in a narrow range around physiological pH. We find that both T3 and PS become less potent with increasing pH, with remarkably similar trends in IC_50_ when T3 is neutral at pH < 7.3. After deprotonation of T3 (but no additional deprotonation of PS) at pH 7.3, T3 loses potency more slowly with increasing pH than PS. We interpret this result as indicating the negative charge is not required for inhibition but does increase activity. Finally, we show that both T3 and PS affect nAChR channel desensitization, which may implicate a binding site homologous to one that was recently indicated for accelerated desensitization of the GABA_A_ receptor by PS.

## Introduction

The nicotinic acetylcholine receptor (nAChR) is an excitatory receptor protein localized in the central nervous system [1], the peripheral nervous system and neuromuscular junction (reviewed in [2]). Pathologies of the receptor, including epilepsy [3] and myasthenia gravis (in muscle-type nAChRs) [4, 5], demonstrate its crucial function in fast synaptic transmission. In muscle-type nAChRs, it is a cation-conducting member of the pentameric ligand-gated ion channel (pLGIC), or “Cys-loop” receptor superfamily [6–10]. Of the five homologous subunits that comprise the nAChR structure and central pore, two are identical (α, γ, α, β, δ). When acetylcholine molecules bind to the α-γ and α-δ subunit interfaces in the receptor’s extracellular domain (ECD), conformational changes propagate to its transmembrane domain (TMD). Four alpha helices (M1-M4) from each subunit form the TMD, with the M2 helices from each subunit lining the receptor’s central pore. Upon binding acetylcholine, the central pore’s inner diameter increases, permitting ion flux through the channel [11].

The pLGIC superfamily also includes glycine receptors, 5-HT_3_ receptors, and γ-aminobutyric acid (GABA_A_) receptors [6], as well as a range of homologs in invertebrates, plants, and prokaryotes [12]. Neurosteroids, either those synthesized in endocrine glands and metabolized, or those synthesized *de novo* in brain tissue, can have hypnotic [13], anxiolytic [14], anxiogenic [15], anti-convulsant [16], and analgesic effects (for review see [17]), and can have synergistic effects with anesthetics [18, 19]. Previous investigations of pLGICs elucidated structure-function relationships of neurosteroids’ molecular features [20–23]. The distinction between molecular features of otherwise similar compounds provides insight into binding sites on the receptor; closely-related molecular species can have differing [22, 24] or opposing [10, 25] effects on their target receptor, and the same neurosteroid can have differing effects on alternative receptor isoforms [26–28].

**Fig 1:**
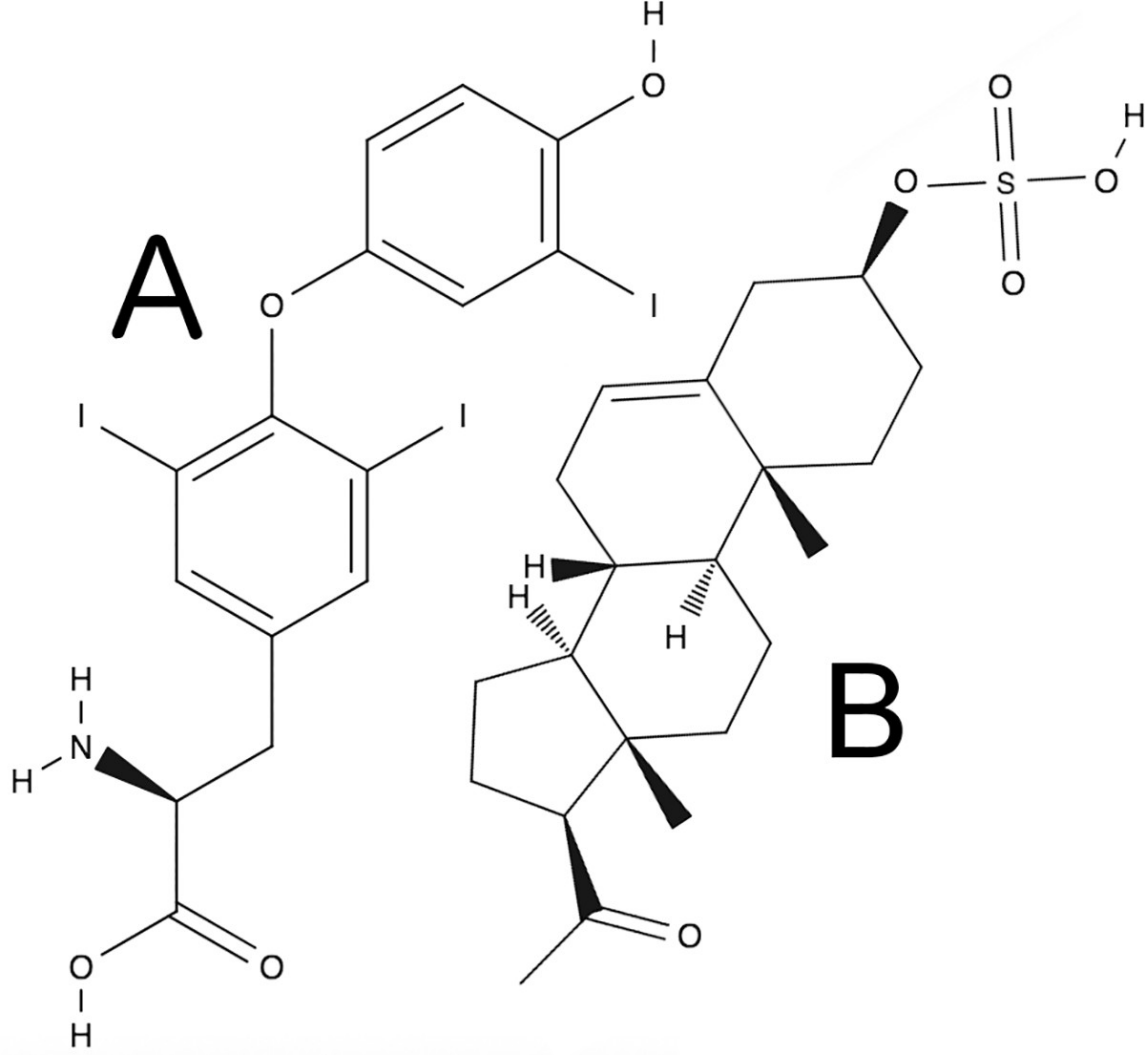
Molecular structure of T3 and PS. Comparisons of structures of the thyroid hormone T3 (A) and the neurosteroid PS (B). T3 and neurosteroids share common features including molecular volume and size, placement of hydrogen-bond accepting groups, presence of carboxylic residues projecting from rings, and charged headgroups.

The neurosteroid 3α-hydroxy-5α-pregnan-20-one (allopregnanolone) activates the GABA_A_ receptor [29], as does the related 5α-pregnane-3α,21-diol-20-one (THDOC) [30–32]. The thyroid hormone L-3,3’,5-L-triiodothyronine (T3) (Fig 1A) was originally theorized to have neurosteroid-like effects due to its similarity to neurosteroids’ size, shape, and lipophilicity [33]. In particular, the results of computational analysis of the overall volumes and shapes of pregnanolone sulfate (PS) and T3 are compared in Figs 5 and 6 of our previous study [33}. Superpositioning of the two molecules showed that not only the molecular volumes, but also the shapes, are nearly identical, and no unmatched functional groups extend from the main axis. Additionally, we have previously published molecular dynamics simulations of single molecules of T3 and allopregnanolone (see S1 Movie and S2 Movie of [29]), illustrating the rigidity of these two molecules in an aqueous solution. The rigidity of the steroid scaffold can be compared to the rigidity of T3 due to the steric hindrance due to the two iodines on the inner aromatic ring of T3.”

Despite structural analogy to positive allosteric modulators, T3 inhibits activity of the GABA_A_ receptor at low concentrations [29, 34–36] and competitively inhibits GABA_A_ receptor activation due to ivermectin or allopregnanolone [29]. The mechanism underlying this surprising result is unknown, and here we test the hypothesis that a population of anionic T3 at physiological pH interacts with pLGICs through a similar mechanism as the anionic neurosteroid pregnanolone sulfate (PS) (Fig 1B). PS is sulfated at C3 but is otherwise similar to positively-modulating neurosteroids, and also inhibits GABA_A_ receptor function [37].

Structural biology has recently provided insight into the binding mode of PS with GABA_A_ receptors. In a recent crystal structure of a homopentameric receptor chimera comprised of a *Gleobacter* ligand-gated ion channel (GLIC) ECD and a mouse GABA_A_R α_1_ TMD, Laverty et al. [38] showed that PS binds alongside a groove between the M3 and M4 helices near the intracellular side of the transmembrane domain of GABA_A_ receptors. This site is involved with the “desensitization gate” recently classified by Gielen et al. [39], indicated by mutations of amino acid residues at the M1-M2 linker and M3 helix of GABA_A_ receptors affected receptor desensitization.

While lipid mixtures used for X-ray crystallography of membrane proteins have become more complex, there are still significant discrepancies with native membranes. pLGICs are extremely lipid sensitive; in particular, they may have specific interactions with anionic lipids [40, 41], polyunsaturated fatty acids [42], and cholesterol [43, 44]. The most feasible pLGIC for study in native membranes is the nicotinic acetylcholine receptor, due to its high concentration in the electric organ of the torpedo electric ray.

Lipophilic modulators often have opposite effects on nAChRs and GABA_A_ receptors; for example, nonhalogenated and halogenated alkane anesthetics inhibit nAChRs but potentiate GABA_A_ receptors (for review see [45]). Most known neurosteroids inhibit nAChRs, regardless of their effect on GABA_A_Rs. Progesterone, dehydroepiandrosterone sulfate (DHEAS), hydrocortisone, and 3α,5α,17β-3-hydroxyandrostane-17-carbonitrile (ACN) inhibit rat neuronal nAChRs [23, 24], while progesterone, estradiol, corticosterone, and dexamethasone non-competitively inhibit human muscle-type [46, 47], ganglionic [46], and *Torpedo* nAChRs [48]. Several lipophilic molecules, including anesthetics and cholesterol, affect both GABA_A_Rs and nAChRs [49–52]. While PS inhibits nAChR-regulated release of catecholamines from the adrenal medulla [53], no effect of PS on nAChR binding was previously seen. Here we report, for the first time, an inhibitory influence of PS on receptor function in muscle-type nAChRs.

Changes in extracellular pH also affect nAChR ion conductance, with more acidic environments yielding lower ion flux and basic environments yielding higher ion flux relative to the receptor’s acetylcholine response at a neutral pH [54, 55]. Different pH environments also affect mean open channel time and desensitization of the receptor [55]. Palma et al. [54] and Li et al. [55] showed that pH changes in the surrounding fluid correlate with changes in nAChR channel conductivity, suggesting that charged residues in the nAChR vestibular and channel pore region affect allosteric transitions to active and inactive states, and that changes in the extracellular pH would titrate these residues. Here we control for the effect of pH on receptor function by comparison of pH effects on modulation by T3 with those of PS, which is not expected to change charge states over the pH range we consider.

We tested the hypothesis that a population of anionic T3 transduces inhibition of nAChR at physiological pH. by extracting functional nAChRs from *Torpedo californica* and used two-electrode voltage clamp (TEVC) to demonstrate functional effects of PS and T3 on nAChRs. We found that both PS and T3 inhibit nAChR activity due to acetylcholine, representing a case in which the same effect on channel function is demonstrated in both nAChRs and GABA_A_ receptors. We also performed TEVC in differing extracellular pH environments to demonstrate the differential effects pH has on ligand charge and resultant receptor activity. Further, we show that PS and T3 modulate the effects of different pH environments on current amplitude when binding acetylcholine. We also show that T3 and PS change nAChR kinetics, increasing desensitization rate at low pHs but minimally affecting desensitization rate at high pHs.

## Materials and methods

### Chemicals

Carbachol, acetylcholine, dimethyl sulfoxide (DMSO), asolectin, T3, NaOH, allopregnanolone, and pregnenolone sulfate were purchased from Sigma Aldrich (St. Louis, MO). Isoflurane was purchased from Henry Schein Animal Health (Dublin, OH). T3 was dissolved in 0.1 M NaOH. Allopregnanolone was dissolved in 0.1% DMSO. All other ligands were dissolved directly in modified Barth’s solution (88 mM NaCl; 1 mM KCl; 0.4 mM CaCl_2_; 0.33 mM Ca(NO_3_)_2_; 0.8 mM MgSO_4_; 5 mM tris(hydroxymethyl)aminomethane-HCl (Tris-HCl); 2.4 mM NaHCO_3_); at low or high pHs, Tris-HCl was replaced with either 2-(*N*-morpholino)ethanesulfonic acid (MES) (T3/PS experiments at pH 6–6.7) or *N*-2-hydroxyethylpiperazine-*N*’-2-ethanesulfonic acid (HEPES) (T3 experiments at 8–9).

### Preparation of crude *Torpedo* nicotinic acetylcholine receptors

All steps before microinjection of resuspended receptor proteins were performed at 0 to 4 °C and were modified from the preparation method described previously [56]. Briefly, flash-frozen *Torpedo* electric organ was purchased from Aquatic Research Consultants (San Pedro, CA). The frozen tissue (600 g) was homogenized in homogenization buffer (10 mM sodium phosphate, 5 mM ethylenediaminetetraacetic acid (EDTA), 5 mM ethylene glycol-bis(β-aminoethyl ether)-N,N,N′,N′-tetraacetic acid (EGTA), 10 mM iodoacetamide, 0.1 mM phenylmethylsulfonyl fluoride (PMSF), 0.02% NaN_3_, pH 7.5) in 30-second bursts at liquefaction setting (Oster blender) and centrifuged for 10 min at 4,080 x g. The supernatant was then filtered through 4 layers of cheesecloth and reserved. The pellets were then resuspended in 200 mL homogenization buffer and centrifugation was repeated. All of the supernatants were then pooled and centrifuged at 134,000 x g for 45 minutes. This pellet was resuspended in 32 mL 28% (w/v) sucrose buffer (10 mM sodium phosphate, 0.1 mM EDTA, 0.02% NaN_3_, pH 7.0). In order to isolate membrane proteins, the aliquots of resuspended pellet (8 mL each) were layered on top of a discontinuous sucrose gradient (10 mL 30% sucrose, 12 mL 35% sucrose, 7 mL 41% sucrose) and centrifuged at 121,800 x g for 4 hours. The membrane band at the interface of the 30–35% sucrose layer was collected using a thin glass transfer pipet, diluted 1:1 with deionized water, and centrifuged at 142,400 x g for 35 minutes. The membranes in the resulting pellet were base-extracted by resuspending them to approximately 0.5 mg/mL protein in water, and the pH was carefully titrated to 11.0 with NaOH, followed by stirring for 45 minutes. The base-extracted membranes were then centrifuged at 142,400 x g for 45 minutes, resuspended in buffer A (100 mM NaCl, 10 mM Tris-HCl, 0.1 mM EDTA, 0.02% NaN_3_, pH 7.4), and flash-frozen in liquid nitrogen.

### Solubilization of crude nicotinic acetylcholine receptor

The protein product from extraction was diluted to 2 mg protein/mL in buffer A. Sodium cholate (10% w/v) dissolved in buffer A was added to give a final cholate concentration of 1%. The mixture was then stirred for 30 minutes, centrifuged at 142,400 x g for 30 minutes, and the supernatant was collected.

### Preparation of asolectin liposomes for resuspension

Solid asolectin lipid was suspended in 4.16% cholate solution in buffer A to form a 65 mM solution. The mixture was vortexed and sonicated at 20 °C under argon gas for 45 minutes, and then stored at 0–4 °C until reconstitution.

### Reconstitution of *Torpedo* nicotinic acetylcholine receptors in asolectin liposomes

Solubilized nAChRs (1–1.65 mg/mL) were mixed with 0.3 mL of liposome/cholate mixture to yield a final volume of 1 mL, and a final concentration of 2% cholate. The mixture was dialyzed for 48 hours against 1,000 volumes buffer A, with a change of buffer every 12 hours. Before use in dialysis, buffer A was bubbled with argon gas for 15 minutes.

### Oocyte microinjection

Ready-to-inject, defolliculated *Xenopus* oocytes were purchased from Ecocyte Bioscience (Austin, TX). The glass injectors were 1.6 to 2 mm o.d., 1.2 to 1.6 mm i.d., and were pulled using a Sutter Instrument Co. Model P-97 puller. The oocytes were injected with 46 nL asolectin-resuspended nAChRs using a digital microdispenser (Drummond Nanoject II). The injected oocytes were incubated at 19 °C in sterile Standard Barth’s solution (SBS; 88 mM NaCl; 1 mM KCl; 0.4 mM CaCl_2_; 0.33 mM Ca(NO_3_)_2_; 0.8 mM MgSO_4_; 5 mM Tris-HCl; 2.4 mM NaHCO_3_) supplemented with 50 mg/L gentamicin.

### Two-electrode voltage clamping of oocytes

Sixteen hours after injection, the oocytes were clamped using a TEVC system. All ligands were dissolved in modified Barth’s solution at the indicated pH and were perfused into the chamber using a gravity-flow system. Thyroid hormones were initially dissolved in 0.1 M NaOH. The oocytes were impaled with two 3 M KCl-filled glass microelectrodes (1–2 MΩ each) and were clamped at -60 mV with an OC-725C Oocyte Clamp (Warner Instruments). Acetylcholine was applied to the bath surrounding the oocyte using a gravity-flow system at 10–13 mL/min. T3 or PS were co-applied with acetylcholine, also using a gravity-flow system. Each ligand perfusion lasted 60–70 seconds; after each perfusion, the bath solution was exchanged with ligand-free buffer until the current response signal had returned to baseline for at least 120 seconds before the next application of ligand(s). Data were recorded using iWorx LabScribe v1.959.

### Data analysis

The current flux signal for each administration of ligand was exported from iWorx into Matlab version 2012b. Due to significant batch-to-batch variation of ion conductance after receptor microinjection, maximal current response (peak) values for inhibition data were normalized to each oocyte's control maximal peak. For all data related to inhibitor concentration curves, the data were fit according to the conditions specified in Table 1. The equation for the fit was Y = Bottom + (Top-Bottom)/(1+10^((LogIC50-X)*HillSlope)).

**Table 1:** Fit types for inhibition-response curves.

Inhibitor/pH	Fit Type	IC50 (μM)	Constraints
T3/PS 7, T3 7.2	log(inhibitor) vs. response	PS 0.77; T3 1.2	Top = 100, Hill Slope = 1
T3/PS 7.5, T3 7.7	log(inhibitor) vs. response	PS 4.6; T3 1.0	Top = 100, Hill Slope = 1
T3/PS 8, T3 8.2	log(inhibitor) vs. response	PS 3.5; T3 1.6	Top = 100, Hill Slope = 1
T3/PS 8.5, T3 8.7	log(inhibitor) vs. response	PS 10.5; T3 4.7	Top = 100, Hill Slope = 1
T3/PS 9, T3 9.2	log(inhibitor) vs. response	PS 2.4; T3 7.3	Top = 100, Hill Slope = 1
Allopregnanolone	log(inhibitor) vs. response—Variable slope	20	None
Triac	log(inhibitor) vs. response	109	Top = 100, Hill Slope = 1

The data were then trimmed to begin with the response as it reached 0.96 of the response peak (to avoid false inflection points by signal noise just after the response’s maximum point), and end 35 seconds post-peak. The data were then fit to a two-degree exponential function (i.e., slow and fast response; $y=a\times e^{-(\frac{x}{A_{1}})}+b\times e^{-(\frac{x}{A_{2}})}$). The slower response of the two-degree exponential function was used in each calculation for analysis of the decay rate, as small irregularities in the raw signal yielded extremely high values for the fast component. For each ligand/environmental condition (n = 3, per condition), decay response and amplitude were calculated and averaged.

## Results

### PS and T3 inhibit nAChRs at concentrations similar to GABA_A_ receptor IC_50_s

**Fig 2:**
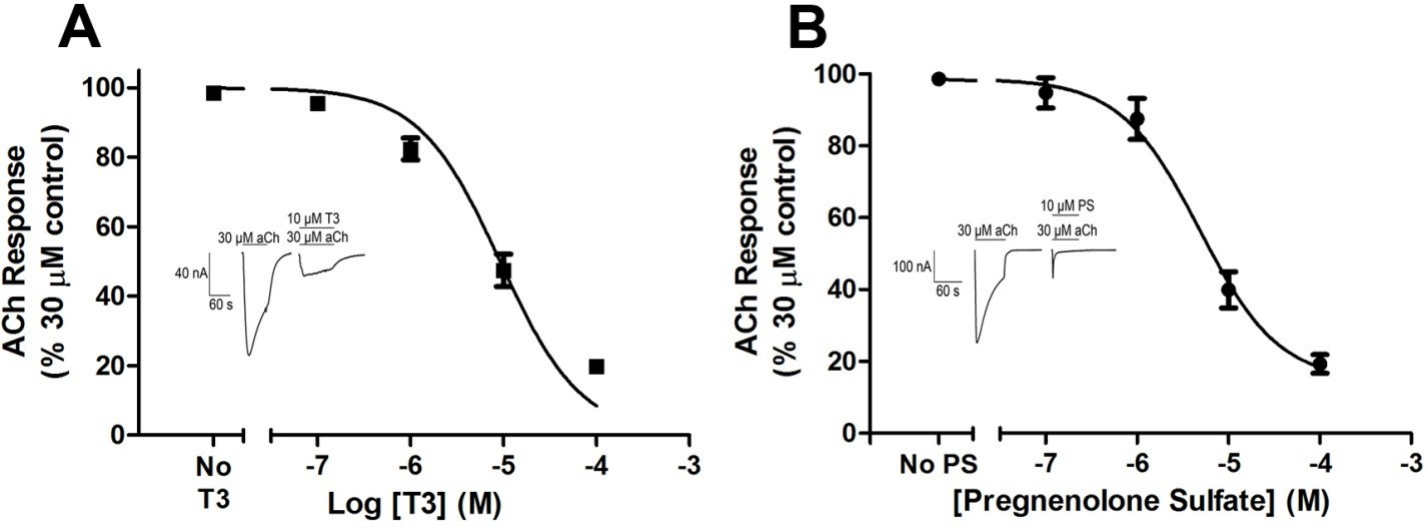
Inhibition of nicotinic acetylcholine receptor response to acetylcholine by T3 and PS. The inhibitory dose-response curves for T3 (A) or PS (B) on ACh-stimulated currents are represented as a percentage of the maximal, non-inhibited response to ACh. Representative tracings show current responses due to application of ACh alone and ACh with T3 (A, inset) or PS (B, inset). Horizontal lines above each trace show the onset and duration of applied ligand(s). The data are represented as mean ± standard error of the mean (SEM). For each data point, n = 3–7.

We co-administered several neurosteroid and neurosteroid-like ligands with acetylcholine during TEVC to determine their effects on nAChR function. Fig 2A shows the inhibitory effect of T3 on nAChR stimulation by 30 μM acetylcholine, as well as a representative trace of the reduction in response due to acetylcholine when co-applied with T3 (Fig 2A, inset). The apparent maximal effect of T3 (seen at ≥ 100 μM) reduced the nAChR control response by 80 ± 6%, with an IC_50_ of 5.4 ± 1 μM T3. This is very similar to the IC_50_ of T3 for the GABA_A_ receptor (8 ± 2 μM) [29].

**Fig 3:**
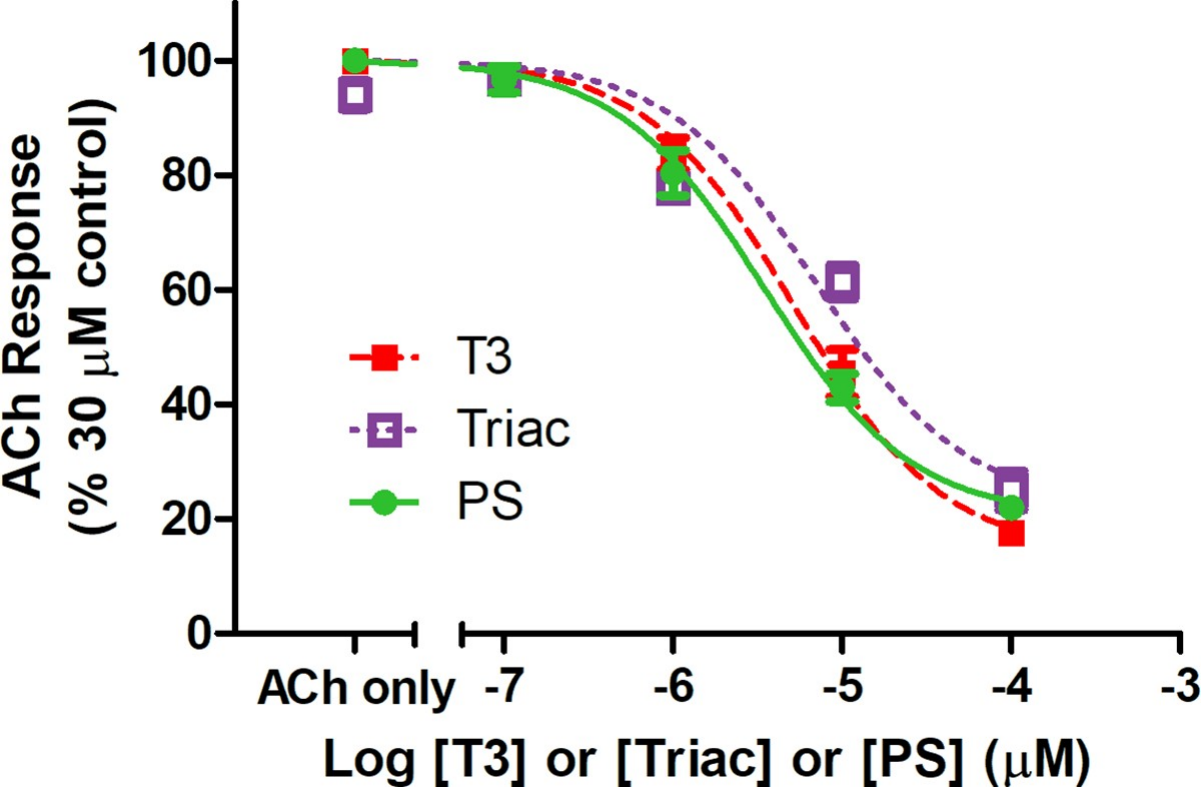
Inhibition of nicotinic acetylcholine receptor response to acetylcholine by T3, PS, and triac. The inhibitory dose-response curves for T3, PS, or triac on ACh-stimulated current, represented as a percentage of the maximal response to ACh. The data are represented as mean ± SEM (n = 3–7).

**Fig 4:**
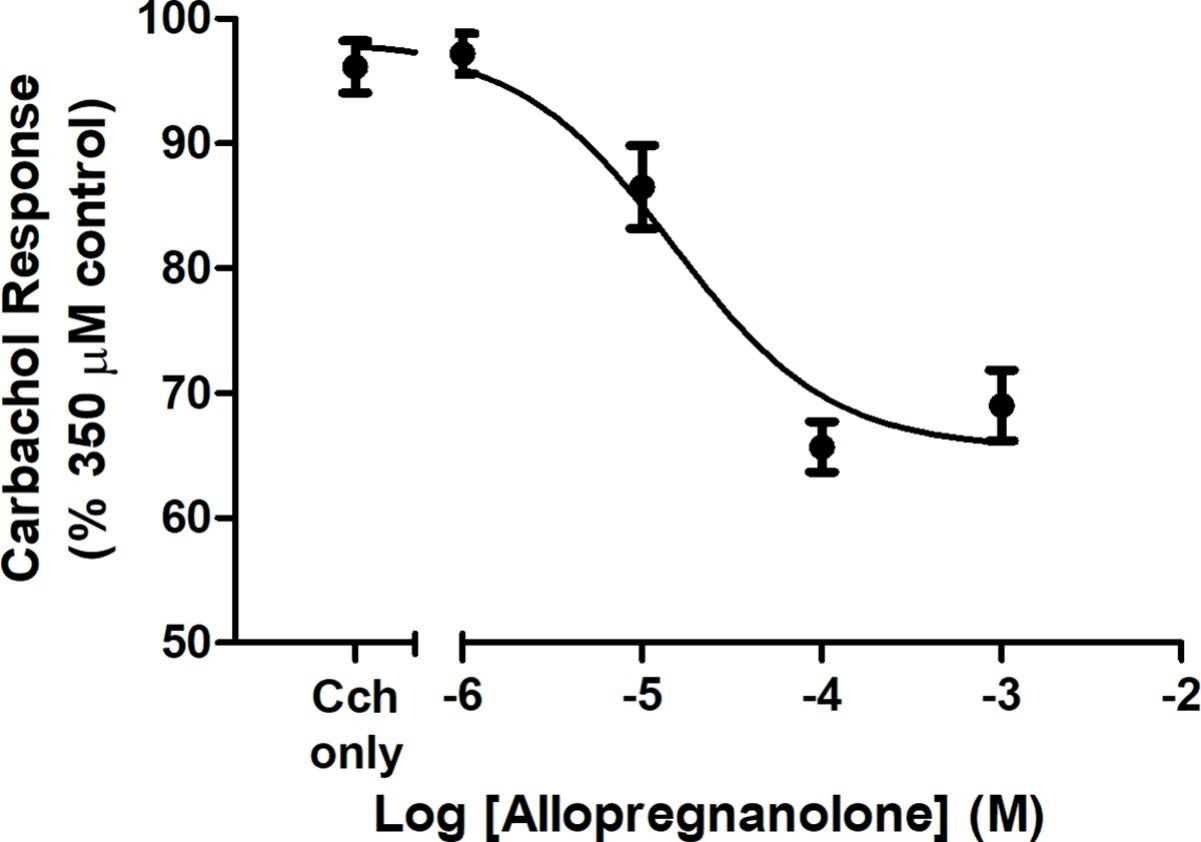
Inhibition of carbachol by allopregnanolone. The inhibitory dose-response curves for allopregnanolone on carbachol (Cch)-stimulated current, represented as a percentage of the maximal response to Cch. The data are represented as mean ± SEM (n = 5).

Fig 2B shows the inhibitory effect of PS on nAChR stimulation by 30 μM acetylcholine, as well as a representative trace of the reduction in response due to acetylcholine when co-applied with PS (Fig 2B, inset). The apparent maximal effect of PS (seen at ≥ 100 μM) reduced the nAChR control response by 81 ± 4%, with an IC_50_ of 4.9 ± 3 μM PS. Triiodothyroacetic acid (triac), which lacks the amine group of T3, also inhibits nAChR, with an IC_50_ of 7 ± 3.1 μM triac (Fig 3). Further, allopregnanolone inhibits activity of nAChRs due to carbachol, an ACh agonist (IC_50_: 20 ± 13 μM) (Fig 4).

### Both negatively-charged and neutral forms of T3 inhibit nAChR

**Fig 5:**
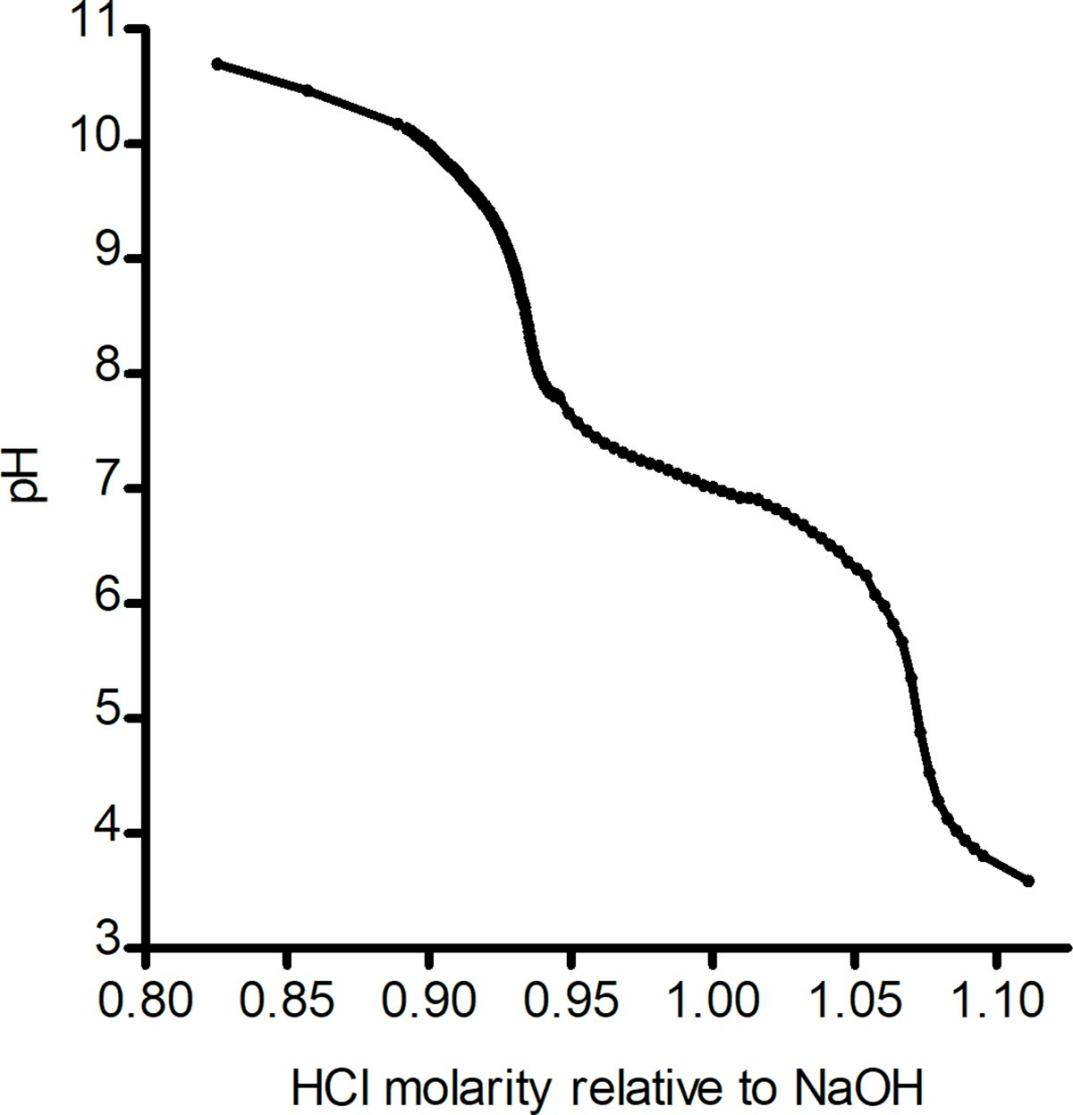
Titration curve of T3. The pK2 value of T3 is ~7.19, corresponding to the deprotonation of the amine group. The (theoretical) pK1 value, corresponding to the carboxylic acid group, is 0.3, while the pK3 value, corresponding to the hydroxy group, is 12.25.

Environmental pH affects molecular charge. According to the titration curve of T3 (Fig 5), T3’s pK2 value is slightly lower than 7.3, in which the hydroxyl group becomes anionic while the amine group remains neutral. However, over this pH interval, PS is expected to retain a negative charge. At physiological pH, there are both charged PS and a proportion of charged T3 molecules. We performed TEVC tests of nAChR inhibition due to T3 at different pHs; changing the surrounding pH allowed observation of effects due to T3’s amino acid (headgroup) in different charge states, as well as effects due to different charge states of the amino acids on the receptor, on nAChR binding. These effects could be compared to the effect due to the unchanging PS sulfate group (headgroup) charge.

**Fig 6:**
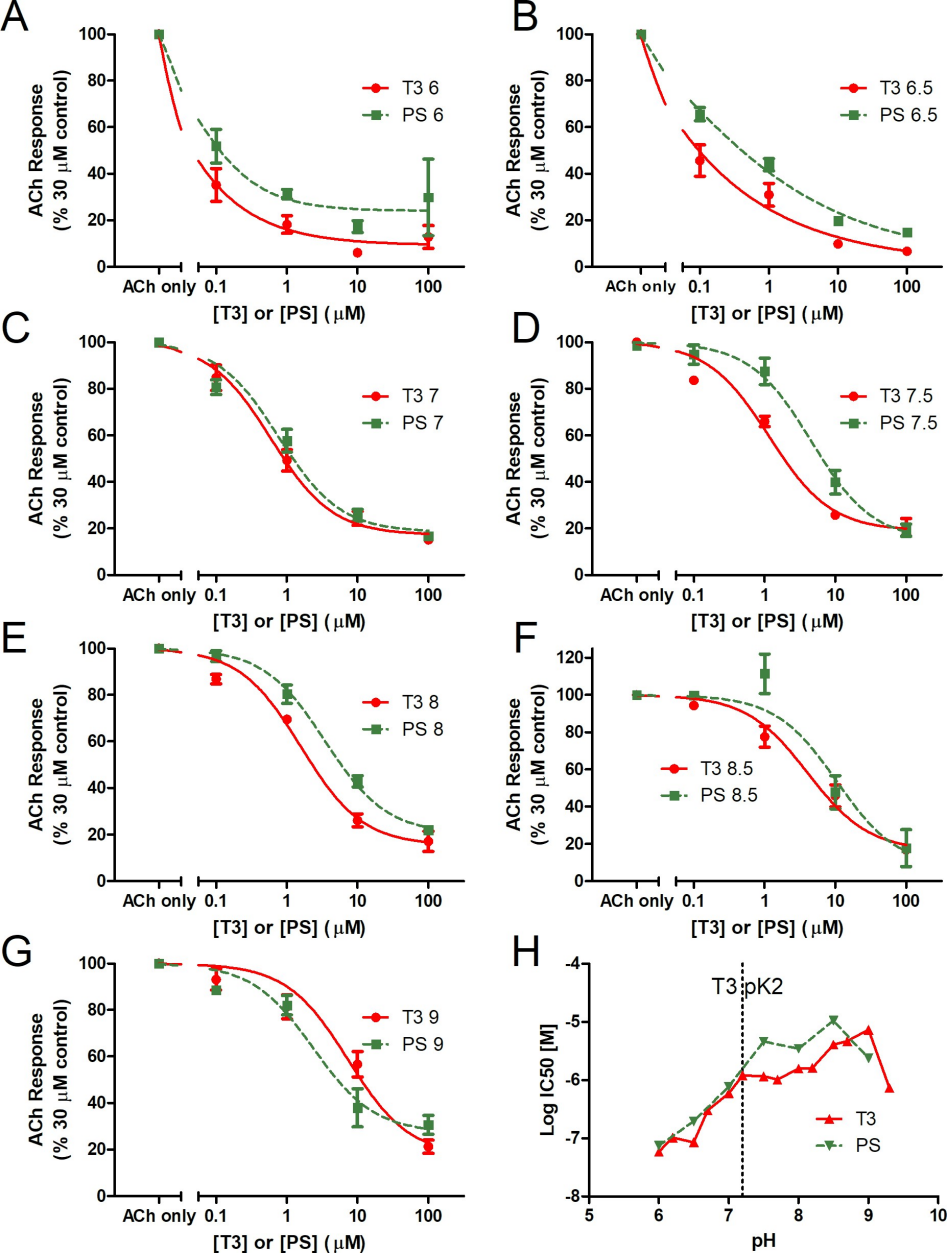
Effects of extracellular pH on inhibition of nAChRs by T3 and PS. The dose-response curves for T3 or PS on ACh-stimulated current were evaluated at pH (A) 6, (B) 6.5, (C) 7, (D) 7.5, (E) 8, (F) 8.5, or (G) 9. Data in (A-G) are represented as a percentage of the maximal response to ACh. The data are represented as mean ± SEM (n = 3). (H) IC_50_ values were generated from the inhibition curves at each pH interval. T3 data is supplemented with inhibition experiments run at each pH level + 0.2 (Fig 7). The dotted vertical line denotes the pK2 value of T3.

The pH environment affects inhibition of nAChRs by both T3 and PS. Dose response curves show the effect of pH (6–9, in 0.5 pH increments) on the IC_50_ of T3 and PS (Fig 6A–6G). A two-way ANOVA (for hormone and dose) indicates that pH has a significant effect on nAChR flux for experiments using T3 (p < 0.0001, F(6, 70) = 74.25) or PS (p < 0.0001, F(6, 70) = 24.02) and that the effect due to the pH environment and due to T3 (p < 0.0001, F(6,70) = 11.69) or PS (p < 0.0001, F(6,70) = 6.214) dose have interactive effects on nAChR flux.

**Fig 7:**
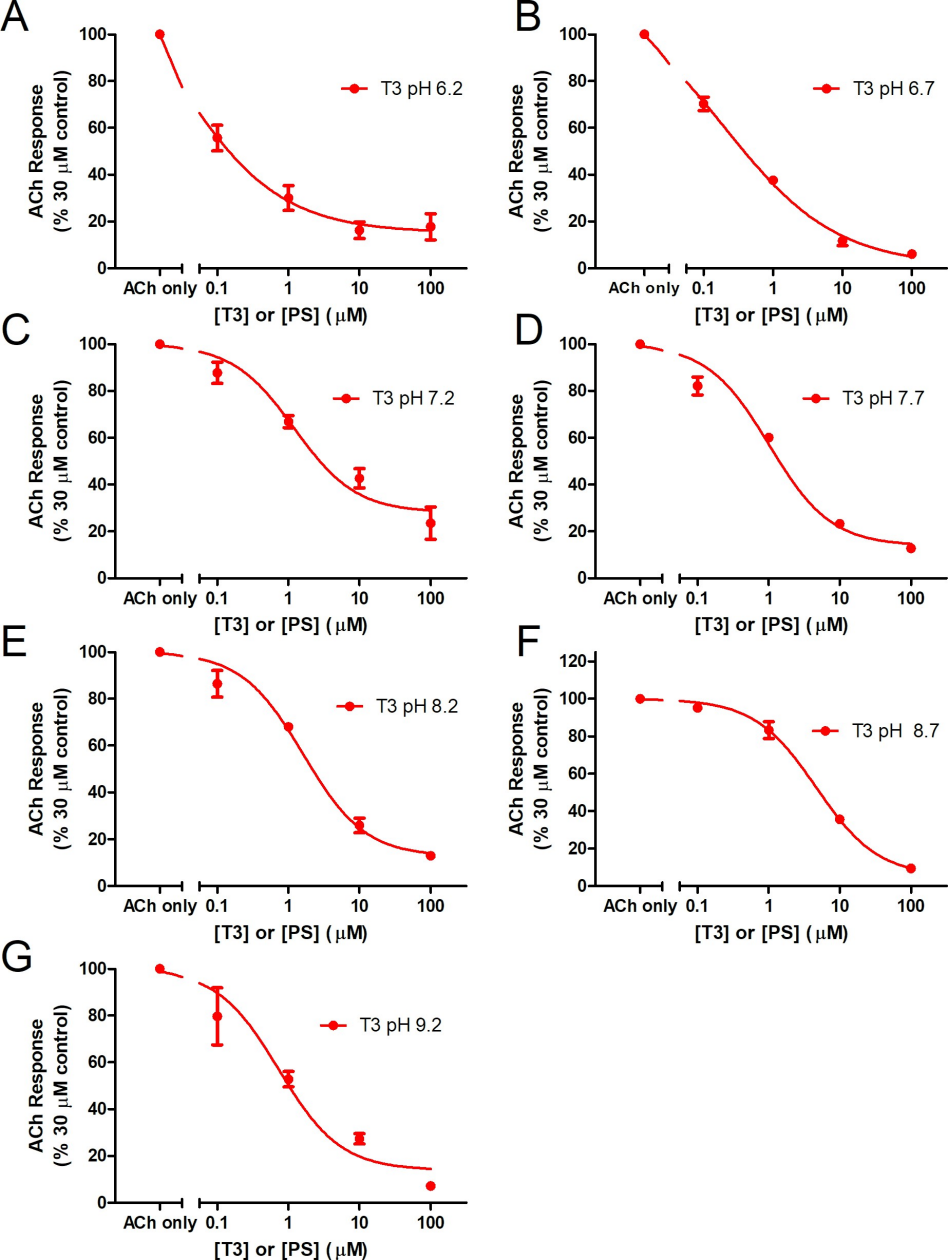
Effects of extracellular pH on inhibition of nAChRs by T3 at offset pHs. The dose-response curves for T3 on ACh-stimulated current were evaluated at pH (A) 6.2, (B) 6.7, (C) 7.2, (D) 7.7, (E) 8.2, (F) 8.7, or (G) 9.2. Data are represented as a percentage of the maximal response to ACh. Error bars represent ± SEM (n = 3).

The effects of pH on inhibition of nAChRs by T3 and PS diverge at the T3 pK2 value. From pH 6–7, the IC_50_ values of T3 and PS (Fig 6H) are similar. However, above the pK2 value of T3 (~7.3), the IC_50_ values of T3 and PS are distinct, with the T3 IC_50_s shifting downward relative to the IC_50_ values of PS. This divergence is coincident with the increase in extracellular pH above the pK2 value of T3. The PS IC_50_ value decreases below T3’s at pH 9, where the net charge of T3 becomes -1, and becomes more prominent than the neutral form dominant at lower pHs.

### T3 and PS affect desensitization kinetics

**Fig 8:**
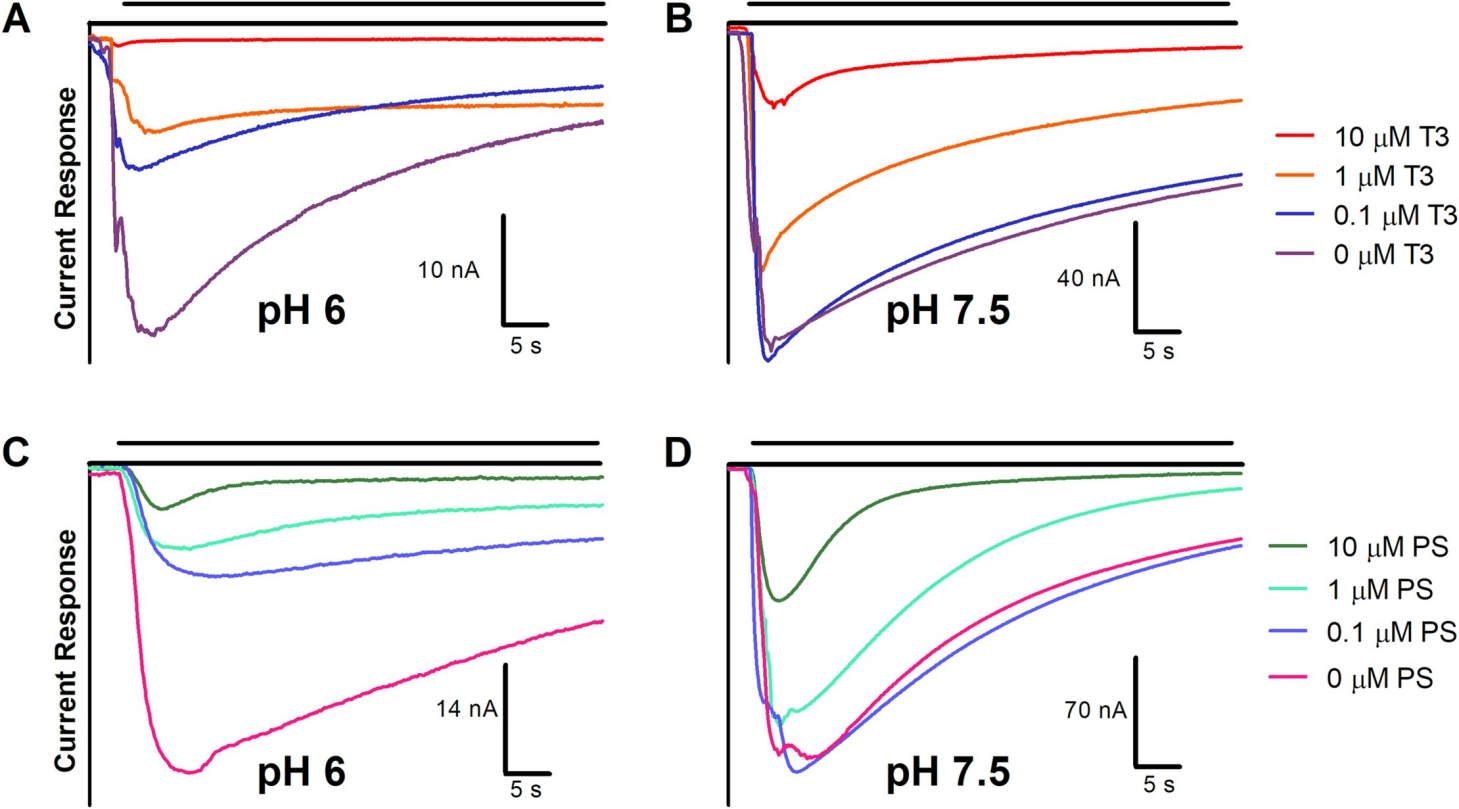
Effect of pH and T3 or PS on nAChR ion conductance. Raw current response traces after administration of 30 μM acetylcholine with the indicated dose of T3 or PS at pH 6 (A, C) or pH 7.5 (B, D). The single, representative traces are close to average values of curve-fit amplitude and decay rate (n = 2–3).

**Fig 9:**
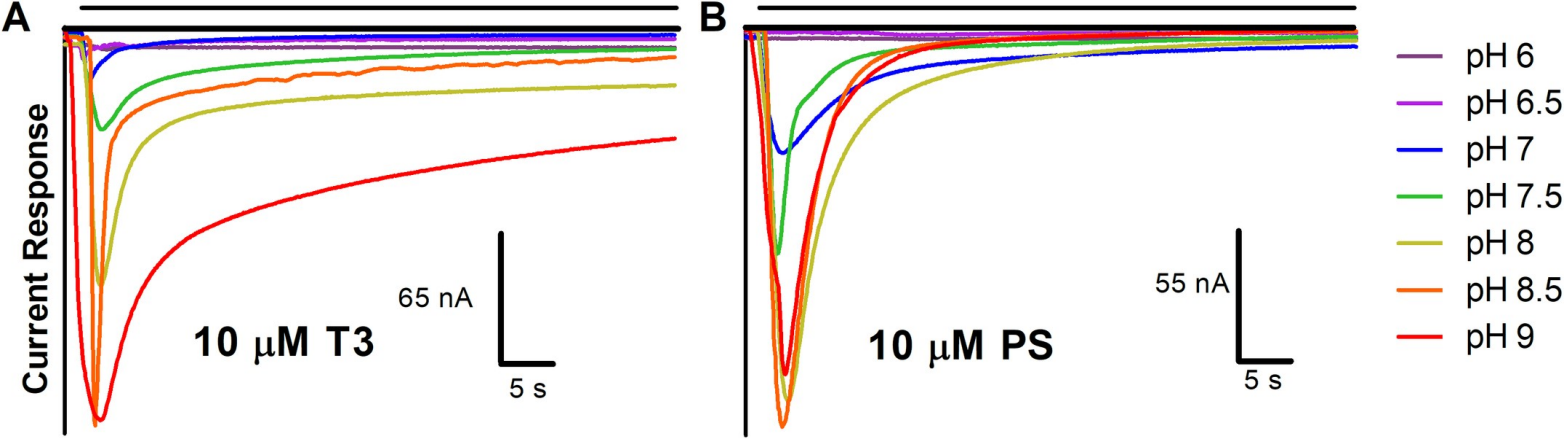
Effect of pH and 10 μM inhibitor on nAChR ion conductance. Raw current response traces after administration of 30 μM acetylcholine with (A) 10 μM T3 or (B) 10 μM PS at differing pH levels. The single, representative traces are close to average values of curve-fit amplitude and decay rate (n = 2–3).

Figs 8 and 9 show representative traces of inhibition due to T3 and PS over 60-second administrations. Raw traces of 0–10 μM T3 or PS at pH 6 and 7.5 (Fig 8) illustrate the effect of dose on the decay rate of ion conductance. According to a two-way ANOVA, dose of inhibitor affects decay rate for both T3 (P<0.0001) and PS (P<0.0001; n = 3, per dose, per inhibitor) (Table 2, Table 3). The representative traces of a 10 μM dose of T3 or PS at each pH (6–9, in 0.5 pH increments) (Fig 9) demonstrate the effect due to ligand dose and pH on desensitization of the channel. According to a two-way ANOVA, at corresponding pHs, PS-inhibited channels exhibit a significantly different decay rate of ion conductance from T3 (P<0.0001) (Table 2). The effect of each ligand on nAChR desensitization may be involved with a region homologous to the desensitization gate structure of GABA_A_ receptors, where PS has been shown to bind [38, 39]. This binding is theorized to cause constriction at the base of the channel pore, inducing a desensitized state [38]. Differences in binding site, orientation or binding affinity may cause differential effects on desensitization.

**Table 2:** Decay Response for T3 and PS.

T3 Decay Response												
	T3 Dose (μM)											
	0			0.1			1			10		
pH	Mean	SEM	N	Mean	SEM	N	Mean	SEM	N	Mean	SEM	N
6	17.7	2.5	2	23.1	2.3	2	10.0	3.2	3	0.4	0.2	2
6.5	26.4	4.2	3	28.0	4.9	3	7.8	0.4	3	1. 2	0.6	2
7	10.8	3.6	3	13.1	1.4	3	9.0	1.4	3	4.2	2.6	3
7.5	22.8	3.0	3	10.2	1.5	3	6.0	0.2	3	2.4	0.5	3
8	23.4	9.5	3	18.8	4.8	3	5.0	1. 2	3	3.6	1.7	3
8.5	12.1	1.0	3	11.7	1.2	3	2.9	0.6	3	1.0	0.3	2
9	5.2	4.1	3	14.8	2.0	3	14.0	7.2	3	13.6	11.0	3
PS Decay Response												
	PS Dose (μM)											
	0			0.1			1			10		
pH	Mean	SEM	N	Mean	SEM	N	Mean	SEM	N	Mean	SEM	N
6	24.3	12.2	3	17.0	10.4	3	8.1	3.8	3	10.5	6.4	3
6.5	30.0	2.6	3	26.2	13.7	3	24.9	15.0	3	11.00	2.8	3
7	28.6	4.2	3	32.7	0.2	3	13.0	6.4	3	4.6	0.3	3
7.5	18.0	9.4	3	32.3	2.2	3	11.9	6.0	3	4.8	1.6	3
8	19.8	3.6	3	20.8	8.8	3	9.4	5.1	3	3.0	0.04	3
8.5	34.0	22.1	3	7.9	7.3	3	1.2	0.6	3	3.2	0.2	3
9	36.8	4.7	3	31.9	1.8	3	18.5	1.5	3	6. 4	1.6	3

**Table 3:** Statistical data for tests of T3 and PS inhibition^a^.

	Factor	Deg of Freedom	F-value	P-value
2-way ANOVA: T3 inhibition decay rate vs. PS inhibition decay rate	Interaction	3	0.9816	0.4031
	Inhibitor	1	16.83	P<0.0001
	Dose	3	25.13	P<0.0001
	Residual	155		
2-way ANOVA: Doses of inhibitor (T3)	Interaction	18	2.068	0.0219
	Inhibitor	3	17.51	P<0.0001
	Dose	6	1.845	0.1088
	Residual	51		
2-way ANOVA: Doses of inhibitor (PS)	Interaction	18	0.6866	0.8094
	Inhibitor	3	11.82	P<0.0001
	Dose	6	1.497	0.1962
	Residual	56		

^a^ 2-way ANOVAs were run for each parameter.

## Discussion

Here we present the first direct observation of T3’s inhibitory effect on nAChRs (Figs 2 and 6). The effect due to T3 on nAChRs is quantitatively similar to the effect due to T3 on GABA_A_ receptors. For the first time, we also demonstrate an inhibitory effect due to PS on nAChR activity (Fig 2B). This effect is comparable to the inhibitory effect due to PS on GABA_A_ receptors [57], and to T3’s effect on nAChRs (Fig 2A) and GABA_A_ receptors [29]. It has been hypothesized that lipophilic ligands have opposing functional effects on GABA_A_ receptors and nAChRs, [45] but here we see this trend does not extend to PS and thyroid hormones.

T3 may act as a neurosteroid-like inhibitor of nAChRs. Thyroid hormone can access the brain via the blood-brain barrier [58–60]; nerve terminal fractions show T3 concentrations of 13.0–65 nM [61, 62], and synaptosomal T3 content is elevated 9.5 times in hypothyroid rats compared when compared to euthyroid rats [63]. Clinical presentations are associated with longer and less restful sleep in hypothyroid patients [64–66], and with insomnia and anxiety in hyperthyroid patients [67, 68]. When injected into the median preoptic nucleus of freely-moving adult rats, T3 increases REM sleep and wakefulness [69, 70], demonstrating short-term, nongenomic effects on behavior. T3 also directly inhibits GABA_A_ receptor activity *in vitro* [29, 34]. The similarity of effects due to T3 and due to neurosteroids, which also modulate sleep [71–74] and anxiety [75, 76], may indicate similar mode of action due to T3 and neurosteroids.

Further, T3 structure resembles the structure of neurosteroids. While it lacks a steroid’s fundamental four-ring molecular group, T3 has a nearly identical volume and shape to PS [33]. Based on molecular dynamics simulations in which the two molecules favorably occupy TM-localized regions, in conjunction with GABA_A_R binding data, Westergard et al. posit that T3 and the PS-like molecule allopregnanolone have a shared binding site in GABA_A_ receptors [29]. This would indicate a possible similar mode of T3 action to that of neurosteroids.

T3 appears to be an exception to established neurosteroid structure-activity relationships. Here we may reject the hypothesis that a population of anionic T3 transduces the inhibitory effect of T3 in nAChR. Further study using analogues of T3 with singular changes in functional groups (e.g., the presence or absence of an amino group, or of bulky and non-bulky groups) may be required to isolate the molecular origin of its unexpected inhibitory effects.

We show here that T3 and PS both affect channel desensitization. Further, changing environmental pH levels appear to affect T3 and PS-induced nAChR desensitization. This may be a result of pH influence on channel residues at the TMD, as has been suggested by previous investigations [54, 55].

The present work adds to the mounting evidence of T3’s nongenomic effect on adult brain tissue [34, 69, 70, 77–80]. These effects may be mediated through interactions with the nAChRs, with GABA_A_ receptors, or with both. This work also indicates, through the inhibitory action of PS and the inhibitory, neurosteroid-like action of T3 on the nAChR, a complex structure-function relationship. The actions of both ligands relative to one another, and to the nearly identical molecules allopregnanolone and triac, can be a tool to investigate binding and inhibition of the channel.
